# The Real Post-Operative Range of Motion Differs from the Virtual Pre-Operative Planned Range of Motion in Reverse Shoulder Arthroplasty

**DOI:** 10.3390/jpm13050765

**Published:** 2023-04-29

**Authors:** Julien Berhouet, Ramy Samargandi, Luc Favard, Céline Turbillon, Adrien Jacquot, Marc-Olivier Gauci

**Affiliations:** 1 CHRU Trousseau Service d’Orthopédie Traumatologie, Faculté de Médecine de Tours, Université de Tours, 1C Avenue de la République, 37170 Tours, France; ramy.samargandi@hotmail.com (R.S.);; 2 Equipe Reconnaissance de Forme et Analyse de l’Image, Laboratoire d’Informatique Fondamentale et Appliquée de Tours EA6300, Ecole d’Ingénieurs Polytechnique Universitaire de Tours, Université de Tours, 64 Avenue Portalis, 37200 Tours, France; 3 Department of Orthopedic Surgery, Faculty of Medicine, University of Jeddah, Jeddah 23218, Saudi Arabia; 4 Centre for Chirurgie des Articulations et du Sport (ARTICS), 24 rue du XXIème Régiment d’Aviation, 54000 Nancy, France; 5 Institut Locomoteur et du Sport, Hôpital Pasteur 2, 30 Voie Romaine, 06000 Nice, France

**Keywords:** reverse shoulder arthroplasty, range of motion, preoperative planning, scapulothoracic joint, soft tissues, motion analysis

## Abstract

Introduction: The purpose of this study was to analyze the real range of motion (RoM) measured in patients operated on for reverse shoulder arthroplasty (RSA) and compare it to the virtual RoM provided by the preoperative planning software. Hypothesis: There was a difference between virtual and real RoM, which can be explained by different factors, specifically the scapula-thoracic (ST) joint. Methods: Twenty patients with RSA were assessed at a minimum follow-up of 18 months. Passive RoM in forward elevation abduction, without and with manually locking the ST joint, and in external rotation with arm at side were recorded. The humerus, scapula, and implants were manually segmented on post-operative CTs. Post-operative bony structures were registered to preoperative bony elements. From this registration, a post-operative plan corresponding to the real post-operative implant positioning was generated and the corresponding virtual RoM analysis was recorded. On the post-operative anteroposterior X-rays and 2D-CT coronal planning view, the glenoid horizontal line angle (GH), the metaphyseal horizontal line angle (MH), and the gleno-metaphyseal angle (GMA) were measured to assess the extrinsic glenoid inclination, as well as the relative position of the humeral and glenoid components. Results: There were some significant differences between virtual and post-operative passive abduction and forward elevation, with (55° and 50°, *p* < 0.0001) or without ST joint participation (15° and 27°, *p* < 0.002). For external rotation with arm at side, there was no significant difference between planning (24° ± 26°) and post-operative clinical observation (19° ± 12°) (*p* = 0.38). For the angle measurements, the GMA was significantly higher (42.8° ± 15.2° vs. 29.1°± 18.2°, *p* < 0.0001), and the GH angle, significantly lower on the virtual planning (85.2° ± 8.8° vs. 99.5° ± 12.5°, *p* < 0.0001), while the MH was not different (*p* = 0.33). Conclusions: The virtual RoM given by the planning software used in this study differs from the real post-operative passive RoM, except for external rotation. This can be explained by the lack of ST joint and soft tissues simulation. However, in focusing on the virtual GH participation, the simulation looks informative. Some modifications between the glenoid and humerus starting positions before running the motion analysis could be provided for making it more realistic and predictive of the RSA functional results. Level of evidence: III.

## 1. Introduction

Restoring range of motion (RoM) is one of the main challenges of reverse shoulder arthroplasty (RSA). Functional outcomes are closely related to bony or prosthetic impingement, especially during adduction and external rotation with elbow at side [1,2]. Scapular notching is one of the consequences of this inferior impingement [3]. 

Three-dimensional (3D) preoperative planning solutions have been developed to improve glenoid implant positioning and optimize RSA biomechanics [4,5]. In a computer study, Berhouet et al. [6] reported that the placement of the glenoid baseplate was more accurate and reliable when using 3D rather than two-dimensional (2D) preoperative visualization. Virtual RoM information can also be provided by the planning software. This helps to guide the surgeon in the choice and placement of the implants and provides a glimpse into the functional outcomes of the surgery [7]. Werner et al. [8] conducted a virtual study with this modelling software to analyze the effect of glenosphere design and size with the aim of reducing the incidence of scapular notching. However, one of the study’s limitations is that the RoM analysis was only based on the glenohumeral joint and bony impingement, without taking into account scapulothoracic (ST) or scapulohumeral movements and soft tissues constraints. 

ST participation is more important for RSA biomechanics than for a normal shoulder [9,10,11]. While the glenohumeral (GH) joint is 2.5 times more active than the ST joint during abduction or forward elevation in normal conditions, the participation ratio (GH/ST) decreases from 1.5 to 1 after RSA, especially over 30° of range [12]. The benefits of preoperative planning, which provides incomplete and potentially mismatched functional information relative to the real conditions, must be questioned [13]. Additionally, the clinical impact of such software can only be correctly addressed if the planned implant positioning is precisely replicated during the real procedure. In practice, this implies that accurate post-operative imaging is needed [14].

**Figure 1:**
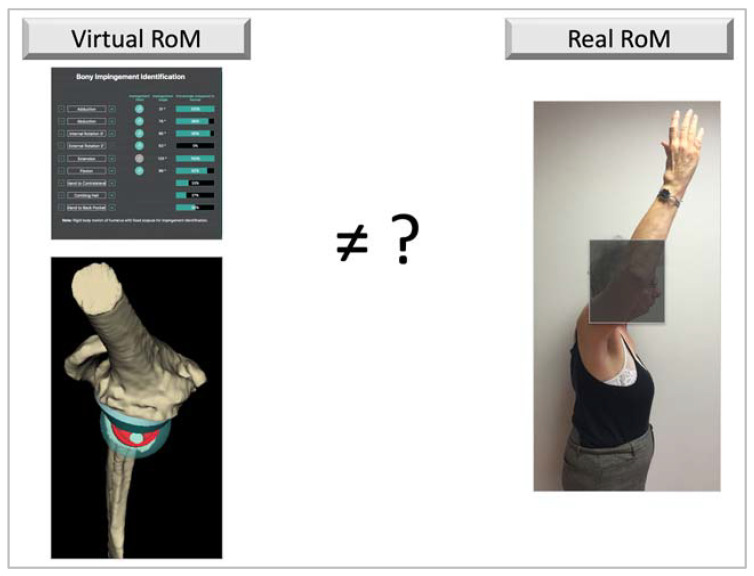
Picture illustrating the goal of the study: comparison of the virtual RoM to the real RoM in case of RSA implantation.

The purpose of this study was to analyze the real RoM measured in patients operated on for an RSA and compare it with the virtual RoM given by the preoperative planning software in the same implant positioning conditions (Figure 1). The hypothesis was that there was a difference between virtual and real RoM, which can be explained by different factors, specifically the ST joint.

## 2. Materials and Methods

### 2.1. Ethics

The study was approved by our institutional review board (IRB: 13B-T-SHOULDER-RM).

### 2.2. Patients and Implants

This was a retrospective clinical and experimental study involving 20 patients (6 males, 14 females), who were operated on for RSA by two surgeons between 1 January and 31 December 2016 (IRB: 13B-T-SHOULDER-RM). The mean age was 73.8 (63–85) years. The mean BMI was 27.1 (19.5–34.4) kg/m^2^. The surgical indications were cuff tear arthropathy (CTA) for 11 patients, primary osteoarthritis (OA) for 7 patients, and rheumatoid arthritis and post-instability arthritis for 2 patients. 

In four patients, the AEQUALIS™ REVERSED II shoulder system was used, while the AEQUALIS™ ASCEND™ FLEX shoulder system was implanted in 16 patients (Wright Medical, Memphis, TN, USA). 

Patients with missing post-operative RoM data and pre- and post-operative imaging were excluded.

### 2.3. D Pre-Operative Planning Software

The BluePrint^®^ 3D Planning software (version 2.1.6, Wright Medical France, Monbonnot Saint Martin, France) provides a validated method for automatic segmentation, 3D reconstruction, and accurate 3D pre-operative glenoid (version and inclination) and humeral (inclination and posterior subluxation) measurements [15,16]. This information can then be used to precisely plan the size and position of the glenoid and humeral implants.

**Figure 2:**
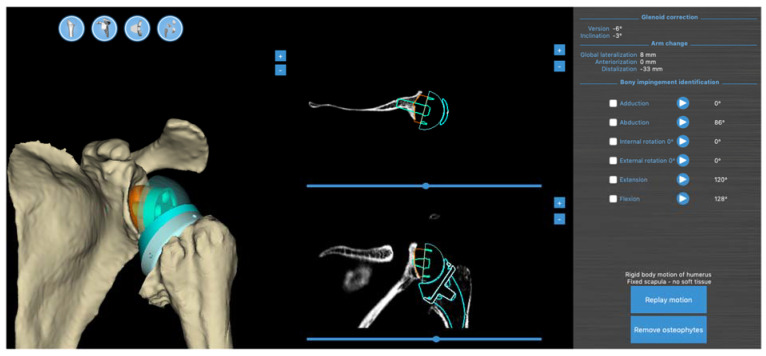
Screenshot of RSA planning with the positioning of the glenoid and humeral components and the virtual RoM analysis results on the right of the picture.

At the end of the planning session for the RSA procedure, the BluePrint^®^ software runs a virtual RoM analysis in the different degrees of freedom: abduction/adduction, flexion/extension, and internal/external rotation arm at side. The maximum range of motion relative to bony or implant impingement between the humeral and scapular sides is recorded (Figure 2). 

### 2.4. Study Protocol

There were seven steps in the protocol.

**Figure 3:**
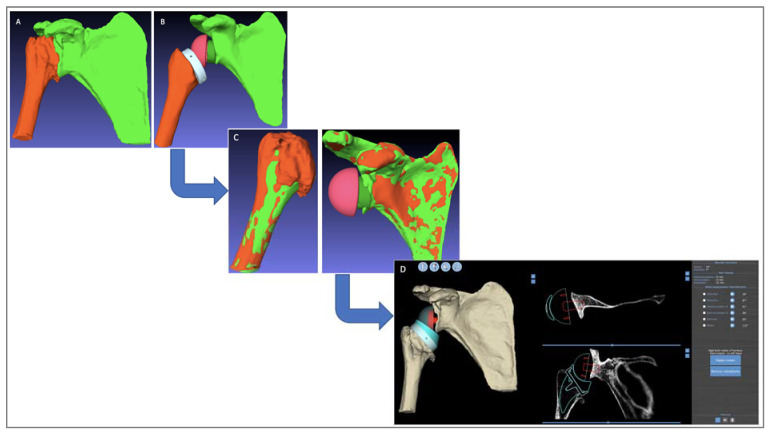
Study protocol. (**A**) Pre-operative automated joint segmentation for extracting humerus and scapula 3D models from the pre-operative CT scans. (**B**) Post-operative manual joint segmentation of the humerus, scapula, and implants from the post-operative CT scan. (**C**) Registration between pre-operative (green) and post-operative (orange) bony structures. (**D**) Post-operative planning with virtual RoM analysis after registration.

1. After the usual pre-operative evaluation (clinical exam, X-ray, and CT scan), the RSA procedure was performed for each patient;
2. At a minimum follow-up of 18 months, a post-operative CT scan was performed for each patient using the acquisition characteristics required by the BluePrint® 3D Planning software, in addition to the routine clinical and radiological assessments, which were conducted by a single surgeon;
3. For all shoulders, the planning software’s automated segmentation algorithm was used to extract the pre-operative humerus and scapula 3D models from the pre-operative CTs (Figure 3A);
4. On all the post-operative CTs, the humerus, scapula, and implants were segmented manually (Figure 3B);
5. Registration and superposition were performed between the pre- and post-operative bony structures using the PTC Creo® Version 6.0 software (Parametric Technology Corporation, Needham, MA, USA) (Figure 3C);
6. A software program that takes the registration data and generates “post-operative planning” files equivalent to the real post-operative implant positioning was developed;
7. Surgeons could open the post-operative planning file and access all BluePrint® 3D Planning software measurements, in particular, the RoM analysis (Figure 3D).

This protocol guaranteed that the implant choice and positioning were identical between the virtual and real conditions, making it possible to compare the RoM measurements between both conditions.

### 2.5. Clinical and Radiological Assessment Criteria

Passive RoM in abduction and forward elevation without and with manually locking the scapula-thoracic joint, as well as the external and internal rotation with elbow at side, were assessed clinically in each patient at a minimum follow-up of 18 months. Those measurements were compared to the virtual RoM generated by the “postoperative planning”. 

**Figure 4:**
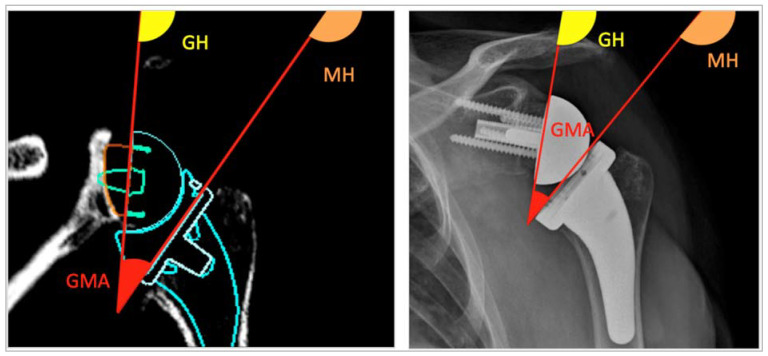
Angle measurements on planning 2D-CT coronal view (**left**) and post-operative anteroposterior X-ray view (**right**). Glenometaphyseal angle (GMA) in red, metaphyseal horizontal line angle (MH) in orange, and glenoid horizontal line angle (GH) in yellow.

Three angles were measured on post-operative anteroposterior x-rays in neutral rotation, and on a planning 2D-CT view passing by the larger diameter of the sphere. The first was the glenometaphyseal angle (GMA) [17] between the prosthetic humeral metaphysis and the backside of the baseplate. The two other angles—metaphyseal horizontal line angle (MH) and glenoid horizontal line angle (GH)—corresponded to the angles between the humeral metaphysis and the horizontal line, and the glenoid component and this latter line, respectively (Figure 4).

### 2.6. Statistical Analysis

The statistical analysis was performed by a biostatistician using XLSTAT Life Sciences software (Addinsoft, Bordeaux, France). The two-sided Wilcoxon–Mann–Whitney test was used to compare two variables. The significance level was set at *p* ≤ 0.05. 

## 3. Results

At a mean follow up of 26.3 months (18–38), the mean post-operative passive abduction was 136° (±28°), while the virtual abduction given by the software was 81° (±13°). There was a significant difference of 55° between the two conditions (*p* < 0.0001). Passive real forward elevation was 141° (±24°), while the virtually planned motion was 91° (±24°). This 50° difference was significant (*p* < 0.0001). 

When manually locking the scapula-thoracic joint, the mean post-operative passive abduction was 65° (±18°), and the passive forward elevation was 63° (±20°). The differences with the planned virtual motions were, respectively, 15° and 27° (*p* < 0.002 and *p* < 0.0001). In those conditions, the abduction ratio (GH/ST) was 0.92. The forward elevation ratio (GH/ST) was 0.81.

For the external rotation with elbow at side, there was no significant difference between the virtual motion (24° ± 26°) and the post-operative clinical observation (19° ± 12° with ST; 22° ± 19° without ST) (*p* = 0.38; 0.42). Internal rotation was not interpretable because the measurement scale differs between the virtual motion given by the software (in degrees) and the real post-operative motion assessed by the surgeon (vertebral level reached) (Table 1).

For the different radiological angles, the GMA was significantly higher on the virtual planning (42.8° ± 15.2°) than on the post-operative X-ray (29.1° ± 18.2°) (*p* < 0.0001). The GH angle was significantly lower on the virtual planning (85.2° ± 8.8° vs. 99.5° ± 12.5°, *p* < 0.0001), while the MH angle was not different (*p* = 0.33) (Table 2).

## 4. Discussion

Post-operative passive forward elevation and abduction were significantly different to the virtual post-operative RoM, while the features and the positioning of the components were exactly the same between planning and surgery. However, those differences were less important when the ST joint was manually neutralized during the clinical evaluation. 

The ST joint was not taken into account during the RoM simulation. In our study, the ratios (GH/ST) for passive forward elevation and abduction in usual clinical exam conditions (ST joint free) are estimated at 0.82 and 0.92. Those values are close to previous values observed by different authors based on kinematic studies [10,12], where a more important participation of the ST joint is reported in case of RSA (1 < ratio (GH/ST) < 1.5) [18]. It could, thus, be considered that GH participation in passive forward elevation and abduction after RSA is quite correctly simulated by the software used in our study. The remaining differences we found between ST locked virtual and real conditions could be explained by the lack of reliability or accuracy of the measurements by the observer, as well as by the soft tissues. The results for the passive external rotation with elbow at side reinforce this interpretation. There was no significant difference between the virtually planned and the real post-operative passive external rotation. The ST joint’s involvement in this motion is different and minimal [19]. In practice, this could mean that passive external rotation is limited by bony inferior scapular impingement, as well as by soft tissues with subscapularis tensioning (12 closing tendon repairs in this study) or fibrosis. Consequently, the virtual RoM analysis generated by the software looks informative for the GH participation rendering in the different passive motions (abduction and forward elevation). It looks even more realistic for external rotation elbow at side, mainly based on GH participation. 

ST joint participation in shoulder RoM partially depends on glenoid inclination. Some authors reported that the more the glenoid is downward, the greater the action of the ST joint for carrying out this motion, especially for abduction and forward elevation [20,21]. For this reason, different angles were measured to investigate the differences between virtual and real conditions. The glenoid inclination analysis may be divided into extrinsic tilt, according to the position of the full scapula, and intrinsic tilt, according to the position of the glenoid joint line relative to the rest of the scapula in the coronal plane. Thanks to the study protocol, the intrinsic glenoid tilt could be considered as the same between post-operative X-rays and virtual planning. However, the GMA was different between those both conditions. This difference came from the GH angle, which assesses the extrinsic and intrinsic glenoid inclination. MH did not change because it reflected the humerus metaphysis inclination, which was not dependent of the scapular inclination. In practice, the software placed every scapula in the same position, with the supraspinatus fossa aligned to a horizontal line. The extrinsic glenoid tilt is systematically neutral in these planning conditions, while it is not the case in reality after surgery. For most of the patients, there is an inferior tilt of the scapula in resting position, which theoretically could prevent inferior impingement and scapular notching, and improve the rotations [22]. Consequently, it could be interesting to place the scapula in the same position as it was pre-operatively during the planning. It could also inform us about the GH joint’s participation, as well as the ST joint’s reserve to carry out certain motions. The conditions of virtual motion would, thus, be closer to the post-operative real conditions.

**Figure 5:**
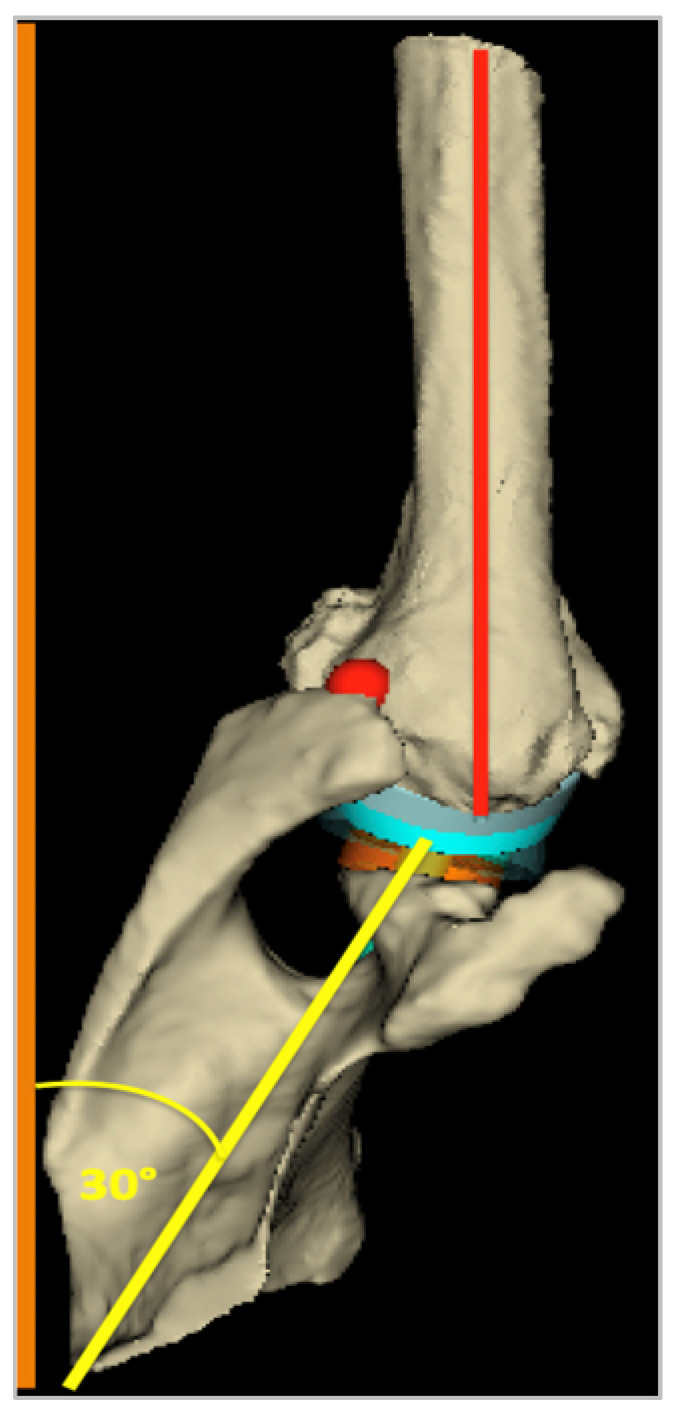
Screenshot of the virtual abduction given by the software (3D superior view). Note the nonaligned position of the humerus (red line) with the scapular plane (yellow line), which makes about a 30° angle with the frontal plane, while the humeral plane is parallel to the frontal plane (orange axis).

Other factors may explain the differences between virtual and post-operative real RoM. The humerus position during simulated abduction is not strictly aligned with the glenoid surface (Figure 5). The abduction motion is consequently not performed in the plane of the scapula, although it aims to be during the clinical exam. It may affect the range of abduction by changing the zones of bony impingement. Thus, the humerus position relative to the glenoid could be modified in the software to optimize the virtual RoM. Soft tissue interposition and tensioning may also limit the RoM before the bony impingement occurs [23]. There is currently no information on how to measure or assess this restriction in the passive motion. 

Our study has several limitations. The number of patients involved is relatively small. The method for measuring the post-operative passive mobilities with the manual neutralization of the ST joint is not so easy achieve and it is not reproducible, and consequently, not so accurate for assessing the range of motion. This could be one of the reasons, as reported above, for explaining the difference observed between virtual and real RoM results. Additionally, this clinical evaluation remains subjective and surgeon-dependent. 

Nevertheless, to our knowledge, this is the first study reported aiming to address the relevance of a pre-operative planning software program with a RoM simulation. Our study protocol was rigorous. The same conditions were used for implant choice and positioning between the planning and the surgery. The passive RoM with and without the ST joint was assessed. Utilizing the limited available data, an associated radiological analysis was produced.

## 5. Conclusions

The virtual RoM given by the 3D planning software programs used in this study differs from the true post-operative RoM, except for the external rotation with elbow at side. This can likely be explained by the lack of simulation rendering for ST joint and soft tissues during the virtual analysis. However, the RoM simulation provided looks informative for GH participation. Some modifications, such as the starting scapula inclination, as well as its position relative to the humerus during abduction, could be inputted in the planning software to make it more realistic and predictive of the RSA functional results.

## Figures and Tables

**Table 1:** Comparison of virtual and real post-operative RoM (GH: gleno humeral joint; ST: scapula thoracic joint). The two-sided Wilcoxon–Mann–Whitney test was used to compare two variables.

	Virtual (1)	Real GH (2)	Real GH + ST (3)	*p*-Value (1 vs. 2)	*p*-Value (1 vs. 3)
Passive Abduction (°)	80.8 ± 13.6	65.6 ± 18.2	136.7 ± 27.7	<0.0001	<0.0001
Passive Forward Elevation (°)	90.8 ± 23.1	63.3 ± 19.7	141.1 ± 23.82	<0.002	<0.0001
External rotation arm at side (°)	24.1 ± 25.6	22.1 ± 19.4	19.2 ± 12.4	0.42	0.38

**Table 2:** Comparison of post-operative X-rays and CT planned angles measurements. The two-sided Wilcoxon–Mann–Whitney test was used to compare two variables.

	Planning	Post-Operative	*p*-Value
Gleno-Metaphyseal Angle (°)	42.8 ± 15.2	29.1 ± 18.2	<0.0001
GH angle (°)	85.2 ± 8.8	99.5 ± 12.5	<0.0001
MH angle (°)	133.3 ± 11.1	128.6 ± 12.3	0.33

## Data Availability

Data supporting reported results can be available in the Department of Orthopedic Surgery of the CHRU Trousseau of Tours, France.
